# Long non-coding RNAs: a rising biotarget in colorectal cancer

**DOI:** 10.18632/oncotarget.14728

**Published:** 2017-01-18

**Authors:** Jian Luo, Jian Qu, Dong-Kai Wu, Zhi-Li Lu, Yue-Sheng Sun, Qiang Qu

**Affiliations:** ^1^ Department of Pharmacy, Xiangya Hospital, Central South University, Changsha, P. R. China; ^2^ Department of Pharmacy, The Second Xiangya Hospital, Central South University, Institute of Clinical Pharmacy, Central South University, Changsha, P. R. China; ^3^ Department of Cardiothoracic Surgery, Xiangya Hospital, Central South University, Changsha, P. R. China; ^4^ Department of Pathology, The Affiliated Cancer Hospital of Xiangya School of Medicine, Central South University, Changsha, P. R. China; ^5^ Department of General Surgery, The Third Clinical College of Wenzhou Medical University, Wenzhou People’s Hospital, Wenzhou, P. R. China

**Keywords:** colorectal cancer, long non-coding RNAs, invasion, epithelial-mesenchymal transition

## Abstract

Colorectal cancer (CRC) is a common gastrointestinal cancer, with a high incidence and high mortality. Long non-coding RNAs (lncRNAs) are involved in the development, invasion and metastasis, early diagnosis, prognosis, the chemoresistance and radioresistance of CRC through interference with mRNA activity, directly combining with proteins to regulate their activity or alter their localization, influencing downstream gene expression by inhibiting RNA polymerase and regulating gene expression as competing endogenous RNAs. Recent progress in next generation sequencing and transcriptome analysis has revealed that tissue and cancer-type specific lncRNAs could be useful prognostic markers. Here, the CRC-associated lncRNAs from recent studies until October 2016 are reviewed and multiple studies that have confirmed CRC-associated lncRNAs are summarized. This review may be helpful in understanding the overall relationships between the lncRNAs involved in CRC.

## INTRODUCTION

RNAs that do not encode proteins are called non-coding RNAs (ncRNA). Those with a length of more than 200 nucleotides are referred to as long non-coding RNAs (lncRNAs) [1]. LncRNAs play important roles in biological processes through interfering with mRNA activity, directly combining with proteins to regulate their activity or alter their localization, influencing downstream gene expression by inhibiting RNA polymerase, and regulating gene expression as competing endogenous RNAs (ceRNAs) [2, 3].

Colorectal cancer (CRC) is the third most commonly diagnosed cancer in humans [4]. CRC is a malignant lesion of the colorectal mucosal epithelium caused by environmental, genetic and epigenetic factors [5]. The genesis of CRC is a multi-stage and multi-gene process, but the mechanism is not yet fully understood. Numerous lncRNAs have been discovered to be involved in influencing gene expression levels via chromatin modification, transcription and posttranscriptional processing [6]. With the discovery of novel molecular and epigenetic mechanisms in CRC, lncRNAs have become a rising biotarget for diagnostic, prognostic, and therapeutic applications. For example, colon cancer associated transcript 2 (CCAT2) underlies metastatic progression and chromosomal instability in colon cancer via the up-regulation of v-myc avian myelocytomatosis viral oncogene homolog (MYC), miR-17-5p, and miR-20a resulting in an enhancement in Wingless and Int1 (WNT) signaling activity [7]. Cancer susceptibility candidate 2 (CASC2) can act as an “miRNA sponges” and ceRNA by sponging miR-18a to upregulate protein inhibitor of activated signal transducer and activator of transcription 3 (PIAS3) and then consequentially inhibit of CRC cell proliferation and tumor growth by extending the G0/G1-S phase transition [8]. Moreover, Long intergenic non-coding RNA 152 (Linc00152) as a ceRNA can confer oxaliplatin resistance via the Linc00152/miR-193a-3p/ erb-b2 receptor tyrosine kinase 4 (ERBB4)/ serine/threonine kinase 1 (AKT) signaling axis and acts as a prognostic indicator in colon cancer patients [9].

Although previous reviews have summarized the CRC-associated lncRNAs, a comprehensive and in-depth analysis of their mechanisms in the invasion, metastasis, early diagnosis, and prognosis of CRC has not yet been reported [10–12]. Herein, the latest papers concerning the 71 CRC-associated lncRNAs reported up to October 2016 are reviewed and multiple studies that have confirmed CRC-associated lncRNAs are summarized. This review may be helpful in understanding the overall relationships between the lncRNAs involved in CRC.

## LNCRNAS INVOLVED IN THE INVASION, METASTASIS, EARLY DIAGNOSIS, AND PROGNOSIS OF CRC

### HOTAIR

HOX transcript antisense RNA (HOTAIR) is the first lncRNA found to demonstrate a trans-transcriptional regulation function and it is located on chromosome 12q13.13. Previous studies have shown that HOTAIR plays an important role in many tumors including prostate cancer, gastric cancer, cervical cancer, and breast cancer [13–16]. HOTAIR interacts with the polycomb repressive complex 2 (PRC2; SUZ12, EZH2, and H3K27me3) and high HOTAIR expression is correlated with the presence of liver metastasis and poor prognosis in CRC patients [17]. Decreasing HOTAIR expression has been shown to inhibit the growth of human CRC stem cells [18]. HOTAIR has potential as a prognostic factor, as it is not only highly expressed in the primary tumors of CRC patients, but is also found in the peripheral blood [19]. HOTAIR can increase the expression of E-cadherin and decrease the expression of vimentin and matrix metallopeptidase 9 to function as a pleiotropic modulator participating in epithelial-mesenchymal transition (EMT), which is involved in CRC invasion, lymph node and organ metastasis, differentiation, vascular invasion and tumor staging [20].

### CCAT

Colon cancer associated transcript 1 (CCAT1) is a recently discovered lncRNA of 2628 nucleotides in length. It is located on chromosome 8q24.21, 515 kb upstream of the MYC oncogene, in a region termed a “super enhancer” [21]. Previous research has shown that CCAT1 is involved in esophageal squamous cell carcinoma, glioma, colorectal carcinoma, and gastric cancer [22, 23]. Recent studies have demonstrated that the level of CCAT1 is significantly higher in the plasma of CRC patients compared with that of healthy controls [24, 25]. Increased plasma HOTAIR and CCAT1 together could be used as a predictive biomarker for CRC [25]. CCAT1 overexpression was associated with CRC proliferation and invasiveness, clinical stage, lymph node metastasis and survival time of CRC [26–28]. The bromodomain and extraterminal (BET) protein bromodomain containing 4 (BRD4) is critical for colon cancer proliferation, and decreasing levels of BRD4 influence differentiation effects during BET inhibition. Therefore, CCAT1 acts as an enhancer-templated RNA, which predicts BET sensitivity in CRC [29].

CCAT2, a novel lncRNA mapping to 8q24, is highly overexpressed in colon cancer and underlies metastatic progression and chromosomal instability in colon cancer [7]. Moreover, previous studies have found that CCAT2 is related with many tumor types such as glioma, gastric cancer, bladder cancer, and small cell lung cancer [30–33]. MYC, miR-17-5p, and miR-20a are up-regulated by CCAT2 through transcription factor 7 like 2 (TCF7L2)-mediated transcriptional regulation and the interaction between CCAT2 and TCF7L2 results in an enhancement in WNT signaling activity, which promotes tumor growth and metastasis in CRC [7].

### MALAT-1

Metastasis associated lung adenocarcinoma transcript 1 (MALAT-1), also called nuclear enrichment autosomal transcript 2, is located on chromosome 11q13.1, has a total length of 8.7 kb and is involved in many types of cancer including pancreatic cancer, liver cancer, gastrointestinal cancer, breast cancer, and lung cancer [34–36]. MALAT-1 is highly expressed in CRC and it can promote the phosphorylation of serine and arginine rich splicing factor 1 (SRSF1), catalyzed by SRSF protein kinase 1-to increase the expression of A-kinase anchoring protein 9, and therefore promotes cell proliferation, invasion and metastasis in CRC [37, 38]. High expression of MALAT-1 has been identified as a biomarker for poor prognosis in CRC [39]. The 3 ‘end of MALAT-1 is an important position in terms of invasion and metastasis in CRC [40]. MALAT1 interacts with Chemokine (C-C Motif) Ligand 5 in tumor-associated dendritic cells to mediate the progression of CRC [41].

### H19

H19 is located on human chromosome 11p15.5 and is a 2.3 kb lncRNA, which constitutes a pair of imprinted genes together with the insulin-like growth factor-II gene (IGF2) [42]. Loss of imprinting of IGF2 in CRC is linked to hypomethylation of H19 and IGF2 [42]. H19 expression has been shown to be involved in solid tumors in a variety of cancers including lung cancer, breast cancer, and gastric cancer [43]. H19 recruits eukaryotic translation initiation factor 4A3 (eIF4A3) to promote CRC proliferation. Moreover, high expression of H19 is associated with tumor differentiation and tumor node metastasis (TNM) staging. H19 is an independent predictor of overall survival and disease-free survival in CRC patients [44]. Gene mutations such as rs2839698 in H19 have been linked with susceptibility to CRC and may function as a potential prognostic factor [45]. H19 was shown to modulate the expression of multiple genes involved in EMT by acting as a ceRNA for miR-138 and miR-200a to influence the migration of CRC [46]. H19 and its product miR-675 are highly expressed in CRC; high levels of miR-675 have been shown to reduce the expression of tumor suppressor retinoblastoma protein (RB) through recognizing and binding the 3′ end of its UTR [47].

### lncRNA-p21

lncRNA-p21 is regulated by p53 to reduce cell viability and its expression is lower in CRC [48]. Moreover, lncRNA-p21 levels in patients with stage III CRC are significantly higher than in those with stage I CRC [49]. lncRNA-p21 reduces cancer cell survival and self-renewal capacity and promotes cancer cell glycolysis via inhibiting the β-catenin signal to inhibit CRC cells with stem cell-like features from developing into mature cancer cells [50]. lncRNA-p21 is also involved in non-small cell lung cancer, gastric cancer, and hepatocellular carcinoma [51, 52].

### GAS5

RNA-growth arrest-specific transcript 5 (GAS5) is located on the chromosome 1q25.1 and its length is 630 nucleotides. GAS5 has been found at different levels of expression in many types of cancer such as non-small cell lung cancer, breast cancer, and gastric cancer [44, 53, 54]. A lower expression of GAS5 is associated with large tumor size, low histological grade and advanced TNM stage and GAS5 expression is an independent predictor for overall survival in CRC patients [55]. The latest literature shows that GAS5 rs145204276 mutation is significantly associated with the susceptibility and progression in CRC, which implies that it contributes to lymphatic metastasis [56]. High expression levels of GAS5 are significantly associated with the future occurrence of liver metastases and poor prognosis in early stage CRC patients [57]. GAS5 is under the control of p53 and it plays an important role in mediating the p53 response to DNA damage [58].

### ANRIL

Antisense non-coding RNA in the INK4 locus (ANRIL) is a natural antisense noncoding RNA and it transcribed from the antisense cluster of the INK4b-ARF-INK4a gene[59]. It is located at Chr9p21.3 and has a 126.3 kb length [60]. ANRIL has been shown to be a prognostic indicator of nasopharyngeal carcinoma, non-small-cell lung cancer, and epithelial ovarian cancer [37, 61]. ANRIL is upregulated in CRC tissues and is associated with survival rate, cell migration and invasion in CRC patients [62, 63]. In CRC cells, ANRIL positively regulates the proliferation in two- and three-dimensional culture in a p15/p16-pRB pathway-independent manner [60].

### UCA1

Urothelial carcinoma associated antigen 1 (UCA1) is a bladder cancer-specific lncRNA, with a total length of 1439 bp and is located in the 19p13.12. It may produce an oncogenic effect related to glucose metabolism [64]. UCA1 is a common molecular marker for lymph node metastasis and prognosis in various cancers including breast cancer, esophageal cancer, and pancreatic cancer [65]. UCA1 is highly expressed in CRC and is involved in tumor cell proliferation, apoptosis and cell cycle progression of tumor and the prognosis of CRC patients [66, 67]. Moreover, a meta-analysis has shown that UCA1 levels are negatively associated with the overall survival time of CRC patients [68]. Therefore, UCA1 has been identified as a predictive biomarker for the prognosis and survival of CRC patients.

### AFAP1-AS1

Actin filament associated protein 1 antisense RNA 1 (AFAP1-AS1) is an antisense RNA gene encoding AFAP1, first identified in esophaguseal and Barrett tumor development, that may function as a potential biomarker to predict the clinical outcome of cancer patients including those with pancreatic ductal adenocarcinoma, esophageal squamous cell carcinoma, and gallbladder cancer [69, 70]. As a carcinogenic lncRNA, it is highly expressed in CRC and is involved in cell proliferation, colony formation, migration and invasion and it is closely related to tumor size, TNM stage and distant metastasis [71, 72]. Knockdown of AFAP1-AS1 inhibits tumor growth and metastasis via the EMT pathway, indicating its potential to serve as an independent prognostic factor for patients with CRC [69, 71, 73].

### TUG1

Taurine up-regulated gene 1 (TUG1) is located at the 22q12.2 and its transcript as a cancer progression related lncRNA, has been found to be involved in the oncogenesis of some tumors. TUG1 promotes cancer metastasis in cancers such as breast cancer, bladder cancer, hepatocellular carcinoma and osteosarcoma [74]. TUG1 regulates the expression of growth-related genes, activates the expression of EMT-associated genes and plays important roles in signal transduction, cell morphology, migration, proliferation and apoptosis in CRC [75]. Inhibition of TUG1 expression blocked the cell migration ability of colon cancer cells [76]. TUG1 is highly expressed in CRC and it indicates a poor prognosis for CRC and promotes metastasis [62].

### HOTTIP

HOXA transcript at the distal tip (HOTTIP) has been recently discovered and is located at the 5 ‘end of HOX clusters. HOTTIP is a novel predictor of lymph node metastasis and survival in non-small cell lung cancer, gastric cancer and pancreatic cancer [77]. HOTTIP is highly expressed in CRC and promotes the growth of CRC partially via silencing of p21 expression [78]. Overexpression of HOTTIP is thought to be an independent poor prognostic factor for CRC patients [79].

### NEAT1

Nuclear-enriched abundant transcript 1 (NEAT1) is a recently identified nuclear-restricted lncRNA, which has two isoforms including 3.7 kb NEAT1_1 and 23 kb NEAT1_2 [80]. NEAT1 has been reported to be involved in ovarian cancer, gastric cancer and breast cancer [81]. NEAT1 is a possible biomarker for diagnostic purposes, tumor recurrence and prognosis in CRC. The high expression of NEAT1 in the tissue and whole blood of CRC patients is associated with tumor differentiation, invasion, metastasis and TNM staging [82].

### BANCR

BRAF-activated lncRNA (BANCR) was originally identified in melanoma cells with 693 bp in length, is located before the repeating cycle of chromosome 9, is crucial for melanoma cell migration and is closely related to the *BRAF* gene V600E mutation [83]. Overexpression of BANCR has been found in many types of cancer such as bladder cancer, esophageal squamous cell carcinoma, and hepatocellular carcinoma [84, 85]. BANCR is highly expressed in CRC and is related with lymph node metastasis and tumor staging [86]. BANCR induces EMT via a mitogen-activated protein kinase kinase/extracellular signal-regulated kinase-dependent mechanism and then enhances G0/G1 cell cycle arrest and apoptosis by regulating p21 [87, 88]. Shi Y, et al. reported that Ets-1 negatively regulates BANCR expression via the deacetylation of H3 histones within the BANCR promoter to influenced a fentanyl-induced mechanism, thereby inhibiting the invasion and migration of CRC cells [89].

### lncRNA-ATB

lncRNA activated by transforming growth factor beta (TGF-β) (lncRNA-ATB) is involved in proliferation and metastasis in a variety of cancers including non-small cell lung cancer, glioma, and renal cell carcinoma and so on [90–92]. Its high expression in CRC patients is significantly associated with greater tumor size, depth of tumor invasion, lymphatic invasion, vascular invasion, and lymph node metastasis [93]. lncRNA-ATB as tumorigenesis suppresses E-cadherin expression and promotes EMT processes during tumorigenesis. Therefore, lncRNA-ATB provides a promising therapeutic biotarget against cancer progression in CRC patients [94].

### ZFAS1

Zinc finger antisense 1(ZFAS1), a newly identified lncRNA, has been reported to be dysregulated in multiple human cancer types including CRC, breast cancer, and gastric cancer [95–97]. The abundance of ZFAS1 expressed in CRC was found to correlate with lymphatic invasion, TNM stage, tumor invasion and metastasis. Patients who had shorter relapse-free survival and overall survival showed an increased expression of ZFAS1, and Cox multivariate analyses imply that ZFAS1 is an independent prognostic factor in CRC patients [98]. ZFAS1 may function as an oncogene in CRC via destabilization of p53 and through interaction with the CDK1/cyclin B1 complex leading to disease progression and apoptosis [95].

### SPRY4-IT1

SPRY4 intronic transcript 1 (SPRY4-IT1), transcribed from an intron of the SPRY4 gene, has been reported to be dysregulated in various cancers including esophageal squamous cell carcinoma, breast cancer, and gastric cancer[99–101]. It may be useful as an independent predictor for overall survival in CRC patients [99]. SPRY4-IT1 is highly expressed in CRC and promotes cell migration and invasion by modifying the EMT pathway [100].

### MEG3

Maternally expressed gene 3 (MEG3) is located at 14q32.2 and is involved in cancer development and metastasis. It is an imprinted gene expressed from the maternal allele with a length of approximately 1.6 kb from DLK1-MEG3 [102]. MEG3 is abnormally expressed in various human cancers, such as hepatocellular carcinoma, bladder cancer, glioma, gastric cancer and CRC [102–105]. Decreasing MEG3 levels could inhibit cell proliferation and predicts a poor prognosis in CRC patients [106]. Lower expression of MEG3 is significantly correlated with low histological grade, deep tumor invasion, and advanced TNM stage [106]. Moreover, the MEG3 rs7158663 AA genotype, not the GA genotype, significantly increases the risk of CRC [107].

### Other lncRNAs involved in CRC

The important roles of the above-mentioned lncRNAs in the invasion, metastasis, early diagnosis, and prognosis of CRC, have been confirmed through multiple studies. However, there are some other new lncRNAs with involvement in CRC, which have emerged in recent studies. ZNF582-AS1 was found to act as a novel tumor-suppressive lncRNA in CRC. Methylation of ZNF582-AS1 is associated with poor survival of CRC patients [108]. Ubiquitin-like plant homeodomain and really interesting new gene finger domain-containing protein 1 (UHRF1) protein associated transcript interacts with and stabilizes the epigenetic factor UHRF1 by interfering with its β-transducin repeat-containing protein-mediated ubiquitination to play a critical role in the survival and tumorigenicity of CRC [109]. Higher levels of lncRNA-uc002kmd.1 result in the regulation of CD44 as a molecular decoy for miR211-3p to enhance cell proliferation in CRC [110]. C-Myc represses the expression of tissue differentiation-inducing non-protein coding RNA (TINCR) through repressing sp1 transcriptive activity and loss of TINCR expression promotes proliferation and metastasis in CRC [111]. Enhanced expression of lncRNA small nucleolar RNAs (Sox2ot), small nucleolar RNA host gene 20 or prostate cancer non-coding RNA 1 promotes cell migration and invasion in CRC [13, 112, 113]. Lower expression of SLC25A25-AS1 promotes proliferation, chemoresistance, and EMT in CRC [11]. RP11-462C24.1, RP1-13P20.6, Prostate cancer-associated ncRNA transcripts 1, promoter of CDKN1A antisense DNA damage activated RNA (PANDAR), and non-coding RNA expressed in aggressive neuroblastoma were identified as the potential novel prognostic markers for CRC [114–118]. PVT-1 or LOC285194 expression levels are an independent risk factor for overall survival in CRC [119, 120]. The expression of ncRuPAR is related to proliferation and metastasis in CRC patients [121]. Loc554202, LOC100287225 were identified to be tumorigenic in CRC [122, 123]. Lnc34a is upregulated in CRC, contributing to epigenetic miR-34a silencing and CRC proliferation [124]. Moreover, many other lncRNAs have been identified to contribute to invasion and metastasis, or aid in early diagnosis and prognosis in CRC via multiple mechanisms. All the CRC-related lncRNAs have been summarized in Table 1.

**Table 1:** The CRC-associated lncRNAs

**lncRNA**	**Effects**	**Expression**	**Mechanisms in CRC from direct evidence**	**PMID**
HOTAIR	Prognostic marker, carcinogenesis	Overexpressed	Modifies EMT pathway; is associated with PRC2 function.	27069543; 24583926; 21862635; 27298568; 24840737
CCAT1	Carcinogenesis, development, invasion and metastasis	Decreased	Is transcribed from the superenhancer cMYC.	27134049; 26752646; 26064266; 25185650; 23594791
CCAT2	Pathogenesis	Overexpressed	Regulates MYC, miR-17-5p, and miR-20a; modifies WNT signaling pathway.	23796952; 27875818
MALAT-1	Proliferation and metastasis	Overexpressed	Promotes the phosphorylation of SRSF1 to increase AKAP-9; interacts with Chemokine (C-C Motif) Ligand 5.	25031737; 25446987; 25025966; 24244343; 27596298; 21503572; 27313790; 26887056
H19	Prognostic biomarker	Overexpressed	Modifies EMT pathway; is a ceRNA for miR-138 and miR-200a; can recruit eIF4A3; mediates methotrexate resistance through activating the WNT/β-catenin pathway; regulates essential Rb-E2F and CDK8-β-catenin signaling.	26989025; 26068968; 19926638; 27596298; 27919747; 27789274
LincRNA-p21	Prognostic biomarker, radiotherapy resistance	Decreased	Is regulated by p53; inhibits the β-catenin signaling pathway.	24012455; 26497997; 24573322
GAS5	Prognostic biomarker	Decreased	Is under control of p53.	25326054; 26634743; 24926850; 27863421
ANRIL	Proliferation and metastasis	Overexpressed	Modifies p15/p16-pRB pathway.	27314206; 26708220
UCA1	Oncogenes and prognostic factors; drug resistance	Overexpressed	Is related to glucose metabolism; inhibits miR-204-5p.	26380024; 27046651; 26238511; 24977734
AFAP1-AS1	Proliferation and metastasis	Overexpressed	Modifies EMT pathway.	27578191; 27261589; 27596298
TUG1	Proliferation, migration	Overexpressed	Modifies EMT pathway.	26856330; 27421138; 27634385
HOTTIP	Progression	Overexpressed	Can silence the expression of p21.	26617875; 26678886
BANCR	Tumorigenesis	Decreased	Modifies EMT pathway via a MEK/extracellular signal-regulated kinase-dependent mechanism; decreases p21.	25928067; 25013510
LncRNA-ATB	Proliferation and metastasis	Overexpressed	Suppresses E-cadherin and promotes the EMT pathway.	26487301; 25750289
ZFAS1	Prognostic indicator, metastasis	Overexpressed	Destabilizes p53 and interacts with the CDK1/cyclin B1 complex.	26506418; 27461820; 27461828
SPRY4-IT1	Prognostic indicator, proliferation and metastasis	Overexpressed	Modifies the EMT pathway.	27391336; 27621655
CLMAT3	Prognostic biomarker, proliferation	Decreased	Its knockdown enhances Cdh1 and results in p27 Kip accumulation via increased Skp2 protein ubiquitination.	26050227; 27391344
PVT-1	Risk factor, proliferation and invasion	Overexpressed	Activates TGF-β signaling pathway and apoptotic signals.	27596298; 24196785
MEG3	Diagnostic and prognostic target	Decreased	Mediates TP53 signaling.	25636452; 27391432; 26934323
NEAT1	Invasion and proliferation	Overexpressed	Plays oncogenic role in colorectal cancer differentiation, invasion and metastasis.	26314847; 26552600
CASC2	Pathogenesis	Decreased	Upregulates PIAS3 by functioning as a ceRNA for miR-18a.	27198161
Loc554202	Tumorigenesis	Decreased	Activation of specific caspase cleavage cascades.	26362196; 27831651
FER1L4	Cell proliferation, migration and invasion	Decreased	Through suppressing miR-106a-5p and depletion of FER1L4, alone or combined with overexpression of miR-106a-5p.	26224446
FEZF1-AS1	Tumorigenesis and progression	Overexpressed	Interacts with hnRNP-K and activates the WNT/β-catenin pathway.	26848625
CASC11	Proliferation and metastasis	Overexpressed	Targets hnRNP-K to activate WNT/β-catenin signaling; c-Myc directly binds to the promoter regions of CASC11 and increases promoter histone	27012187
SnaR	5-FU-resistance	-	SnaR loss decreases sensitivity to 5-FU.	25078450
SLC25A25-AS1	Proliferation, chemoresistance	Decreased	Promotes EMT process associated with Erk and p38 signaling pathway activation.	27553025
ZNF582-AS1	Diagnostic biomarker	Methylation	Methylation of ZNF582-AS1.	27215978
TINCR	Proliferation and metastasis	Decreased	Loss of TINCR promotes hydrolysis of EpCAM and then release of EpICD, subsequently, activates the WNT/β-catenin pathway.	27009809
UPAT	Tumorigenesis	Overexpressed	Interacts with and stabilizes the epigenetic factor UHRF1 by interfering with its β-transducin repeat-containing protein (TrCP)-mediated ubiquiti	26768845
HULC	Prognostic indicator	Overexpressed	Interacts with EZH2 to repress underlying target NKD2 transcription.	27496341
DACOR1	Tumorigenesis	Overexpressed	Induction of DACOR1 leads to the activation of tumor-suppressor pathways and attenuation of cancer-associated metabolic pathways.	26307088
CCAL	Progression and chemotherapy resistance	Overexpressed	Induces multidrug resistance (MDR) through activating WNT/β-catenin signaling by suppressing AP-2α and further upregulating MDR1/P-gp ex	25994219
cir-ITCH	Tumorigenesis	Decreased	Increases the level of ITCH, which is involved in the inhibition of the WNT/β-catenin pathway.	26110611
ucoo2kmd.1	Proliferation	Overexpressed	Regulates CD44 as a molecular decoy for miR211-3p.	26974151
CTD903	Prognostic indicator	Overexpressed	Downregulates CTD903 enhanced WNT/β-catenin activation and increases transcription factor (Twist and Snail) expression.	27035092
LINC01133	Prognostic biomarker	Decreased	Is downregulated by TGF-β, which could inhibit EMT and metastasis; inhibits EMT and metastasis by directly binding to SRSF6.	27443606
Lnc34a	Proliferation	Overexpressed	Directly targets miR-34a; recruits Dnmt3a via PHB2 and HDAC1 to methylate and deacetylate the miR-34a promoter simultaneously.	27077950
Sox2ot	Proliferation and metastasis	Overexpressed	Cyclin B1 and Cdc 25C are downregulated by knockdown of Sox2ot; decreases N-cadherin, increases E-cadherin.	27353770
CRNDE-h	Prognostic indicator	Overexpressed	Is correlated with IRX5 mRNA expression.	27042112; 27888803
ENST00000430471	Proliferation and invasion	Decreased	Cis-regulation and trans-regulation of co-expressed gene.	27217770
HIF2PUT	Migration, invasion	Overexpressed	Is a regulator of HIF-2α.	26648739
AK027294	Proliferation and metastasis	Overexpressed	Is associated with the regulation of caspase-3, caspase-8, Bcl-2, MMP12, MMP9, and TWIST.	26820130
Linc00152	Prognostic indicator, drug resistance	Overexpressed	Is ceRNA to modulate miR-193a-3p, and then ERBB4; contributes to L-OHP resistance at least partly through activating the AKT pathway.	27633443
CTNNAP1	Diagnostic biomarker	Decreased	ceRNA cross-talk between pseudogene CTNNAP1 and its cognate gene CTNNA1.	27487124
PANDAR	Prognostic indicator	Overexpressed	Modulates the EMT pathway through inhibiting N-cadherin, vimentin, β-catenin, Snail and Twist expression and increasing E-cadherin.	27629879
GAPLINC	Proliferation and metastasis	Overexpressed	Binds to PSF/NONO and partly stimulates SNAI2.	27259250
SNHG20	Proliferation, invasion and migration	Overexpressed	Modulates a series of cell cycle-associated genes.	27543107
91H	Prognostic indicator	Overexpressed	Unclear	25058480
ADAMTS9-AS2	Prognostic indicator	Decreased	Unclear	27596298
BCAR4/HOXA-AS2	Prognostic indicator	Overexpressed	Unclear	27596298
AK123657/BX64820	Prognostic marker	Decreased	Unclear	24809982
ENST00000465846	Lymph node metastasis	Decreased	Unclear	25009386
AK307796/ENST00	Lymph node metastasis	Overexpressed	Unclear	25009386
CAHM(LOC100526)	Tumorigenesis	Decreased	Unclear	24799664
DANCR	Prognostic factor	Overexpressed	Unclear	26617879
DQ786243	Proliferation and metastasis	Overexpressed	Unclear	26934980
FTX	Prognostic biomarker	Overexpressed	Unclear	26629053
GHET1	Proliferation and metastasis	Overexpressed	Unclear	27131316
HOTAIRM1	Prognostic biomarker	Decreased	Unclear	27307307
LINC01296	Prognostic biomarker	Overexpressed	Unclear	25894381
LOC100287225	Pathogenesis	Decreased	Unclear	27062707; 26429648
LOC285194	Prognostic indicator	Decreased	Unclear	23680400
ncRAN	Prognostic indicator	Decreased	Unclear	24519959
ncRuPAR	Proliferation and metastasis	Decreased	Unclear	25119598
NR_029373/NR_034	Prognostic indicator	Decreased	Unclear	27591862
PCAT-1	Prognostic indicator	Overexpressed	Unclear	23640607
PRNCR1	Proliferation	Overexpressed	Unclear	26530130; 24330491
RP1-13P20.6	Biomarker	Decreased	Unclear	27596299
RP11-462C24.1	Prognostic marker	Decreased	Unclear	24954858; 27683121
XLOC_006844/LOC	Tumorigenesis	Overexpressed	Unclear	26328256

Abbreviations: CRC, colorectal cancer; lncRNAs, long non-coding RNAs; ceRNA, competing endogenous RNAs; HOTAIR, HOX transcript antisense RNA; EMT, epithelial-mesenchymal transition; MMP-9, matrix metallopeptidase 9; MALAT-1, metastasis associated lung adenocarcinoma transcript 1; HOTAIR, human homeobox transcript antisense RNA; PRC2, polycomb repressive complex 2; CCAT1, colorectal cancer associated transcript 1; CCAT2, colorectal cancer associated transcript 2; BET, bromodomain and extraterminal; GAS5, RNA-growth arrest-specific transcript 5; snoRNAs, small nucleolar RNAs; siRNAs, small interfering RNAs. IGF2, Insulin-like growth factor-II gene; eIF4A3, eukaryotic translation initiation factor 4A3; NEAT2, nuclear enrichment autosomal transcript 2; AKAP-9, A-kinase anchoring protein 9; SRSF1, serine and arginine rich splicing factor 1; SRPK1, SRSF protein kinase 1; TNM, tumor node metastasis; RB, Retinoblastoma protein; ANRIL, Antisense non-coding RNA in the INK4 locus; UCA1, Urothelial carcinoma associated antigen 1; AFAP1-AS1, Actin filament associated protein 1 antisense RNA 1; TUG1, Taurine up-regulated gene 1; HOTTIP, HOXA transcript at the distal tip; NEAT1, Nuclear-enriched abundant transcript 1; BANCR, BRAF-activated lncRNA; lncRNA-ATB, Long non-coding RNA-activated by TGF-β; ZFAS1, Zinc finger antisense 1; SPRY4-IT1, SPRY4 intronic transcript 1; MEG3, Maternally expressed gene 3; PHD, Ubiquitin-like plant homeodomain; SNHG20, small nucleolar RNA host gene 20; PRNCR1, prostate cancer non-coding RNA 1; PCAT-1, Prostate cancer-associated ncRNA transcripts 1; 5-FU, 5-fluorouracil; CCAL, Colorectal cancer-associated lncRNA; Linc00152, Long intergenic non-coding RNA 152; MDR, multidrug resistance.

### LncRNAs involved in chemoresistance and radioresistance in CRC

Several types of genetic and epigenetic regulatory mechanisms have been identified as involved in the development of chemoresistance in cancer cells. Changes in the expression of lncRNAs are also responsible for resistance to chemotherapy and radiotherapy in cancer. The specific identities and roles of lncRNAs in treatment resistance remain to be more fully elucidated.

LncRNA-UCA1 decreased the 5-fluorouracil (5-FU) chemosensitivity in CRC by attenuating apoptosis via inhibiting miR-204-5p [125]. The UCA1-miR-204-5p-CREB1/BCL2/RAB22A regulatory network plays an important role in pathogenesis and chemoresistance in CRC patients [125]. Down-regulation of lncRNA snaR decreased cell death after 5-FU treatment, which indicates that snaR loss increased the resistance against 5-FU in CRC [126]. Knockdown of SLC25A25-AS1 has been shown to enhance chemoresistance and promotes the EMT process associated with Erk and p38 signaling pathway activation in CRC [11]. Linc00152 acting as a ceRNA of miR-193a-3p increases the levels of ERBB4 contributing to oxaliplatin chemosensitivity in colon cancer [127]. Colorectal cancer-associated lncRNA (CCAL) was identified as modifying the response to adjuvant chemotherapy in CRC patients by inducing multidrug resistance (MDR) through activating WNT/β-catenin signaling by suppressing AP-2α and further upregulating MDR1/P-gp levels [128]. H19 has been shown to mediate methotrexate resistance via activating the WNT/β-catenin pathway, and therefore H19 could be a promising therapeutic target for methotrexate-resistant CRC [129].

Radiotherapy is a standard preoperative treatment approach for local advanced cancer to reduce local recurrence [130]. The down-regulation of HOTAIR has been shown to improve the radiosensitivity of CRC cells [131] and lncRNA-p21 can enhance CRC radiosensitivity by targeting the WNT/β-catenin signaling pathway [130].

### The mechanisms of CRC-associated lncRNAs

Although numerous CRC-related lncRNAs have been identified, to date, little is known of their mechanisms of action. The known mechanisms have been summarized in Table 2. lncRNAs nearly affect the whole life cycle of genes, from transcription to RNA splicing, degradation and translation, and regulate gene expression via diverse mechanisms [11]. From healthy cells to tumor cells and metastases, and even in chemotherapy and radiotherapy resistance, lncRNAs are involved in every aspect of CRC. They can act not only as the major transcription factor but also as one of the co-regulatory factors; they are not only involved in EMT and WNT pathway but also can interact with miRNAs.

**Table 2:** The CRC-associated lncRNAs with different pathways or interactions

**Pathway or interactions**	**lncRNAs**
EMT pathway	HOTAIR, H19, AFAP1-AS1, TUG1, BANCR, lncRNA-ATB, SPRY4-IT1, SLC25A25-AS1, LINC01133, PANDAR, lncRNA-ATB, Sox2ot
WNT pathway	CCAT2, CASC11, TINCR, CCAL, cir-ITCH, CTD903, H19
Interaction with miRNAs	CCAT2, H19, UCA1, CASC2, FER1L4, ucoo2kmd.1, Lnc34a, Linc00152, CTNNAP1
Silencing of p21 expression	HOTTIP, BANCR
Involved with p53	ZFAS1
TGF-β signaling pathway	PVT-1, LINC01133
Erk and p38 pathway	SLC25A25-AS1

Abbreviations: EMT, epithelial-mesenchymal transition; lncRNAs, long non-coding RNAs; HOTAIR, HOX transcript antisense RNA; AFAP1-AS1, Actin filament associated protein 1 antisense RNA 1; TUG1, Taurine up-regulated gene 1; BANCR, BRAF-activated lncRNA; SPRY4-IT1, SPRY4 intronic transcript 1; lncRNA-ATB, Long non-coding RNA-activated by TGF-β; CCAT1, colorectal cancer associated transcript 1; CCAT2, colorectal cancer associated transcript 2; CCAL, Colorectal cancer-associated lncRNA; Linc00152, Long intergenic non-coding RNA 152; ZFAS1, Zinc finger antisense 1

EMT is an important step in cancer development, which involves the cooperation of a variety of signaling pathways including the transformation growth factor-β, Sonic Hedgehog, and WNT pathways [132, 133]. As shown in Table 2, HOTAIR, H19, AFAP1-AS1, TUG1, BANCR, lncRNA-ATB, SPRY4-IT1, SLC25A25-AS1, LINC01133, PANDAR, lncRNA-ATB and Sox2ot wereare involved in the EMT pathway. These lncRNAs could increase the levels of E-cadherin, vimentin, ZEB1, ZEB2, and MMP9, all of which are core marker genes for mesenchymal cells.

Moreover, CCAT2 [7], H19 [129], CASC11 [134], TINCR [111], CCAL [128], cir-ITCH [82], and CTD903 [135] are involved in the WNT pathway, which also regulates gene expression changes during EMT[111]. WNT signaling can promote cancer progression-associated EMT processes via β-catenin-mediated increased gene expression at the invasive front of colorectal tumors [136].

**Figure 1:**
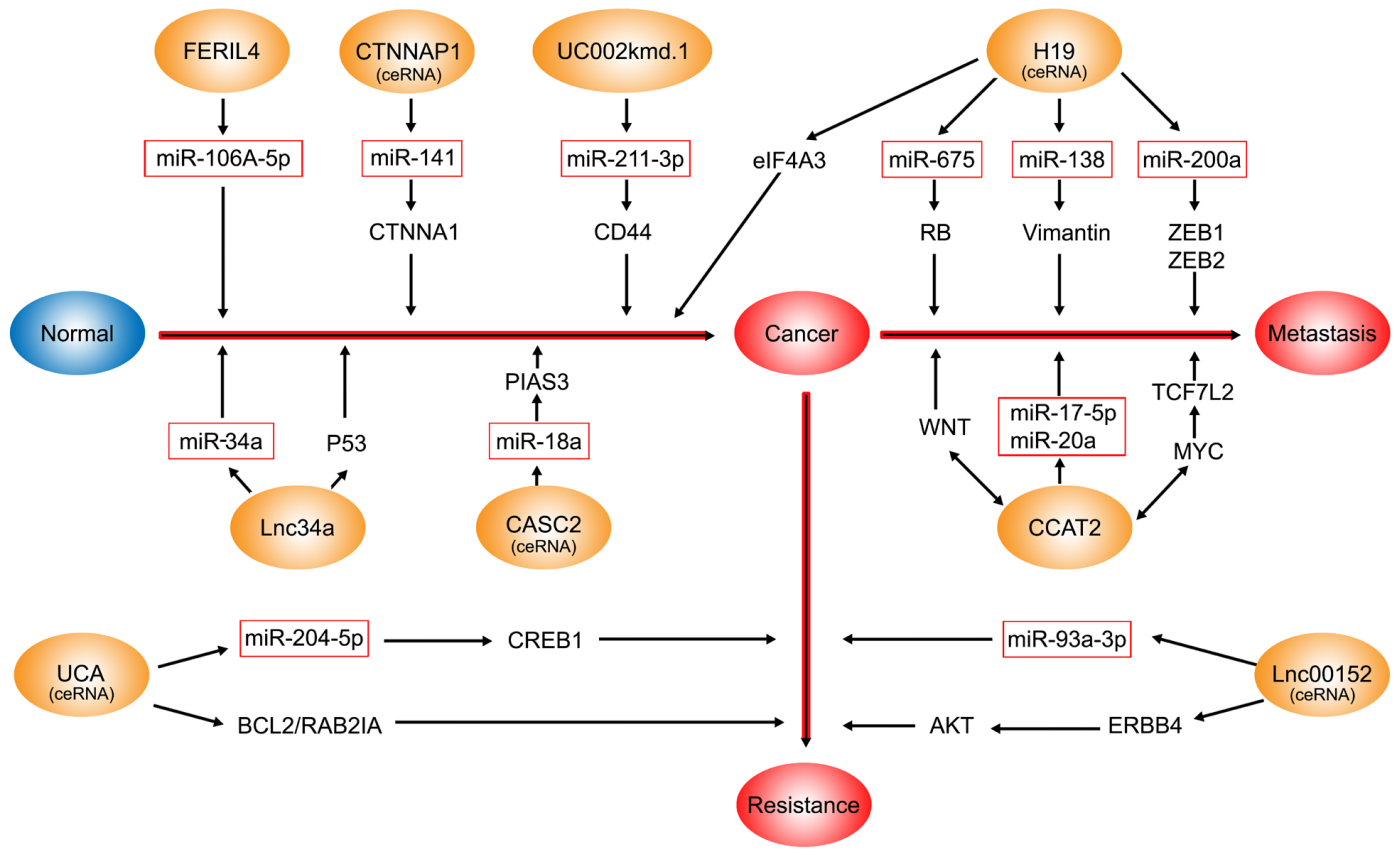
The CRC-associated lncRNAs which interact with miRNAs The blue oval represents normal; the orange ovals represent lncRNAs involved in the interaction with miRNAs; the red rectangles represent miRNAs. FER1L4, CTNNAP1, CASC2, uc002kmd.1, and lnc34a interact with miRNAs to contribute to the pathogenesis of CRC. H19 and CCAT2 are involved in the metastasis and proliferation of CRC via multiple mechanisms including functioning as ceRNAs and through the WNT signaling pathway. Moreover, H19 also promotes tumor growth by recruiting and binding to eIF4A3. The UCA1-miR-204-5p-CREB1/BCL2/RAB22A regulatory network plays an important role in chemoresistance in CRC. lnc00152 acting as a ceRNA of miR-193a-3p increases the levels of ERBB4, contributing to oxaliplatin chemosensitivity in colon cancer.

The CRC-associated lncRNAs that interact with miRNAs are as follows and are shown in Figure 1: CCAT2 [7], H19 [46], UCA1 [125], CASC2 [8], FER1L4 [137], uc002kmd.1 [110], Lnc34a [138], Linc00152 [9], CTNNAP1 [139]. As ceRNAs and “miRNA sponges”, lncRNAs can antagonize miRNA function by inhibiting their endogenous targets, thereby imposing an additional level of post-transcriptional regulation [114].

Other lncRNAs have different mechanisms to carry out their functions (Table 1, Table 2). For example, HOTTIP and BANCR potentially contribute to CRC cell growth partially through the silencing of p21 expression [78, 88]. ZFAS1 may function as an oncogene in CRC via destabilization of p53 [95]. It is not surprising that the action of lncRNAs in CRC does not involve a single mechanism, but a variety of mechanisms co-exist. For example, H19 can function as a ceRNA for miR-138 and miR-200a to influence the migration of CRC cells and H19-derived miR-675 regulates the RB tumor suppressor in CRC [46, 47]. However, H19 recruits eIF4A3 to promote CRC proliferation [44].

## CONCLUSIONS AND FUTURE PERSPECTIVES

lncRNAs play important roles in invasion and metastasis, early diagnosis, prognosis, and chemoresistance and radioresistance in CRC. Large-scale genomic studies using chips to investigate the abnormal expression profiles of lncRNAs in tumors have been carried out and a variety of cancer-related lncRNAs have been reported. However, the molecular mechanisms of these dysregulated lncRNAs remain poorly characterized. Here, all the publications concerning the relationships between lncRNAs and CRC have been reviewed and summarized. As shown in Table 1, Table 2 and Figure 1, there are more than 71 CRC-associated lncRNAs that have been found to date and clear mechanisms have been reported for some of these. However, the full mechanisms underlying all the CRC-associated lncRNAs are currently not understood. Therefore, further functional studies investigating how these related lncRNAs impact on all the processes in CRC in cells, animal models and human clinical trials are needed to advance this research.

Although recent advancements in technology have enabled the rapid development of research into CRC-related lncRNAs, enormous challenges still exist. Firstly, the low levels of some lncRNAs in body fluid or tissues necessitate the use of advanced and reliable protocols of lncRNA amplification and enrichment. Secondly, finding highly specific and sensitive biomarkers from the many CRC-associated lncRNAs is also a huge challenge because many lncRNAs are also involved in the development, invasion, metastasis, chemoresistance and radioresistance of other types of cancer. Moreover, determining the significance of specific lncRNAs in CRC and identifying the real protagonists is a challenge, considering the impact of confounding factors such as race, subject numbers, and TNM. However, despite such challenges, there is reason for optimism.

## Abbreviations

CRC: colorectal cancer
lncRNAs: long non-coding RNAs
ceRNA: competing endogenous RNAs
EMT: epithelial-mesenchymal transition
MALAT-1: metastasis associated lung adenocarcinoma transcript 1
HOTAIR: human homeobox transcript antisense RNA
PRC2: polycomb repressive complex 2
CCAT1: colorectal cancer associated transcript 1
CCAT2: colorectal cancer associated transcript 2
BET: bromodomain and extraterminal
GAS5: RNA-growth arrest-specific transcript 5
snoRNAs: small nucleolar RNAs
siRNAs: small interfering RNAs
IGF2: Insulin-like growth factor-II gene
eIF4A3: eukaryotic translation initiation factor 4A3
SRSF1: serine and arginine rich splicing factor 1
ncRAN: non-coding RNA expressed in aggressive neuroblastoma
TNM: tumor node metastasis
RB: Retinoblastoma protein
ANRIL: Antisense non-coding RNA in the INK4 locus
UCA1: Urothelial carcinoma associated antigen 1
AFAP1-AS1: Actin filament associated protein 1 antisense RNA 1
TUG1: Taurine up-regulated gene 1
HOTTIP: HOXA transcript at the distal tip
NEAT1: Nuclear-enriched abundant transcript 1
BANCR: BRAF-activated lncRNA
lncRNA-ATB: Long non-coding RNA-activated by TGF-β
ZFAS1: Zinc finger antisense 1
SPRY4-IT1: SPRY4 intronic transcript 1
MEG3: Maternally expressed gene 3
UHRF1: finger domain-containing protein 1
5-FU: 5-fluorouracil
CCAL: Colorectal cancer-associated lncRNA
Linc00152: Long intergenic non-coding RNA 152
MDR: multidrug resistance
